# Genotyping bovine leukemia virus in dairy cattle of Heilongjiang, northeastern China

**DOI:** 10.1186/s12917-019-1863-3

**Published:** 2019-05-29

**Authors:** Changqing Yu, Xuefeng Wang, Yulong Zhou, Yu Wang, Xianfeng Zhang, Yonghui Zheng

**Affiliations:** 1 State Key Laboratory of Veterinary Biotechnology, Harbin Veterinary Research Institute, Chinese Academy of Agricultural Sciences, Harbin, China; 2 College of Animal Science and Technology, Heilongjiang Bayi Agricultural University, Daqing, China; 3 Department of Microbiology and Molecular Genetics, Michigan State University, East Lansing, USA

**Keywords:** Bovine leukemia virus, Genotyping, Cattle, Enzootic bovine leukosis

## Abstract

**Background:**

Bovine leukemia virus (BLV) causes enzootic bovine leukosis in cattle and leads to heavy economic losses in the husbandry industry. Heilongjiang Province, China, is rich in dairy cattle. However, its current BLV epidemiology and genotypes have still not been evaluated and confirmed. In this report, we investigated the BLV epidemiology in dairy cattle in the major regions of Heilongjiang Province via the nested PCR assay.

**Results:**

A total of 730 blood samples were collected from nine different farms in six regions of Heilongjiang. The results showed that the infection rate of these regions ranged from null to 31%. With a clustering analysis of 60 published BLV *env* sequences, genotypes 1 and 6 were confirmed to be circulating in Heilongjiang. Importantly, a new genotype, 11, and a new subgenotype, 6E, were also identified in the Harbin and Daqing regions, respectively. An epitope analysis showed that a cluster of T-X-D-X-R-XXXX-A sequences in genotype 11 gp51 neutralizing domain 2 was unique among all currently known BLV isolates and was therefore a defining feature of this new genotype.

**Conclusions:**

BLV epidemics and genotypes were initially investigated in dairy cattle of Heilongjiang. A relatively high infection rate was found in some regions of this province. A new genotype, G11, with a highly specific motif, was identified and thus added as a new member to the current BLV genotype family. This report provides an initial reference for future investigations and subsequent control of BLV transmission and spread in this region.

**Electronic supplementary material:**

The online version of this article (10.1186/s12917-019-1863-3) contains supplementary material, which is available to authorized users.

## Background

Bovine leukemia virus (BLV) is closely related to human T-lymphotropic virus, both of which belong to the *Deltaretrovirus* genus [1, 2]. BLV infection can cause enzootic leukosis in cattle [2, 3] and lead to a reduction in milk production and quality and to decreased longevity in dairy cattle [4–7]. Recently, a correlation between human breast cancer and BLV infection was proposed [8–11], demonstrating that BLV might directly threaten public health. Therefore, these observations suggest an urgent need for surveillance and control of BLV infection.

**Fig. 1:**
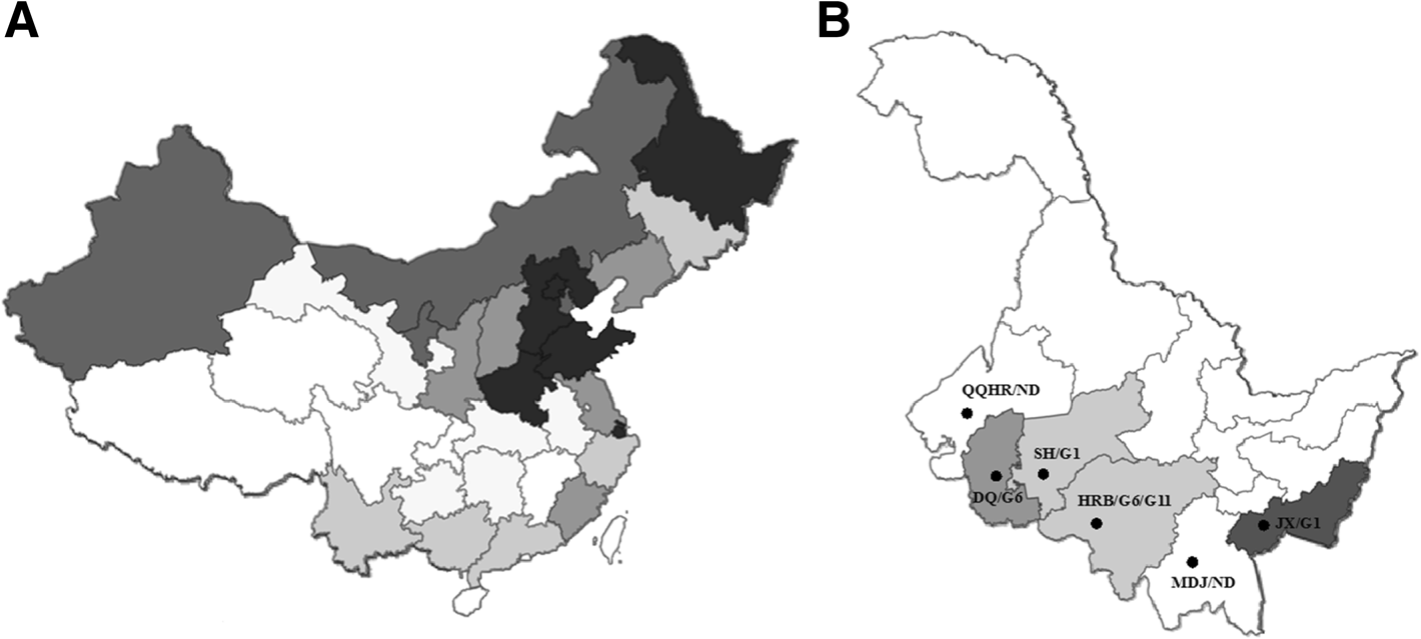
Map of dairy cattle distribution in China and HLJ Province territory. **a** Current dairy cattle distribution in China. Colors from light to dark indicate that the amounts of cattle gradually increased. Cattle amounts in the darkest region were more than 200,000, as in HLJ. **b** The map of HLJ Province territory. We collected samples from six major regions of HLJ. Each region is indicated with a black dot from left to right. Harbin is the capital of HLJ Province. Colors from light to dark indicate that the infection rate gradually increased. QQHR, Qiqihar; DQ, Daqing; SH, Suihua; HRB, Harbin; MDJ, Mudanjiang; JX, Jixi; and ND, not determined

The BLV provirus has approximately 8200 base pairs, which encode four major structural and enzymatic proteins (Gag, Pro, Pol, and Env), two regulatory proteins (Tax and Rex), and two accessory proteins (R3 and G4) [12]. Other than these viral genes, the BLV provirus also contains a microRNA-encoding region [13]. The Gag protein can be cleaved into three parts, termed the matrix protein (MA, p15), the capsid protein (CA, p24), and the nucleocapsid protein (NC, p12) [14]. The Env protein is expressed as a precursor gpr72, which is processed into surface (SU) gp51 and transmembrane (TM) gp30 [14–16]. Based on the env gene polymorphism in the gp51 region, BLV is now classified into 10 genotypes (genotype 1 to genotype 10, G1–G10), which are distributed worldwide [17–35]. BLV genotypes have been reported in countries near China, such as the Philippines [28], Korea [29], and Mongolia [30]. Recently, BLV infection of cattle in China was investigated [5]. Another study reported the genotype prevalence in yaks of southwestern China [36]. However, the BLV genotypes of cattle in other parts of China are still unknown. Heilongjiang Province (HLJ), northeastern China, as one of the largest provinces in the territory, has a strong husbandry industry. HLJ ranks 4th among Chinese provinces with the most dairy cattle (more than 200,000), according to information released by the Dairy Data Center of China (Fig. 1a). Nevertheless, the current status of BLV epidemics in HLJ is unclear, and the BLV genotypes circulating in this region still need to be identified.

Accordingly, we performed an initial investigation of BLV epidemiology in dairy cattle in six major regions of HLJ. We were able to detect BLV proviral DNA in samples from four of these regions. After sequencing, we found that G1, G6, and a new genotype, G11, were prevalent in HLJ. Combined with recent domestic reports [5, 36], our results indicated that BLV infection is common in China, where G1, G6, G10, and G11 are the currently known BLV genotypes.

## Results

### Nested PCR assay for BLV epidemics in major regions of HLJ

Our nested PCR results showed that BLV epidemics existed in the regions of SH, DQ, HRB, and JX, whereas the status varied somewhat among these regions (Table 1). JX had the highest infection rate of 31%. DQ, HRB, and SH are in close proximity with each other, and the infection rates among these regions were similar, with infection rates of 10, 8, and 5%, respectively. Notably, the conventional nested PCR identified an infection rate of 10% in DQ, whereas the CoCoMo-qPCR-2 detection showed more sensitivity with 12.5% infection. No BLV provirus DNA was detected in samples from QQHR and MDJ. As shown in Fig. 1b, QQHR is adjacent to DQ, whereas MDJ is geographically near JX. Although a relatively high BLV infection rate was found in JX and DQ, no BLV infection was detected in QQHR and MDJ, which might suggest strict local management of dairy cattle in both QQHR and MDJ. Taken together, these results indicate a common BLV infection in partial regions of HLJ.

**Table 1:** Detection of BLV infection in six regions of HLJ

Year	Region	Farm	Breed	Age	Samples	Ne-PCR	Cq-PCR	Rate
2015	JX	A/B	Simmental	3–6	110	34	–	31%
2015	DQ	C/D	Holstein	2–5	160	16	20	10%
2015	QQHR	E	Holstein	2–4	100	0	–	0%
2015	HRB	F/G	Holstein	2–4	100	8	–	8%
2015	MDJ	H	Holstein	2–4	60	0	–	0%
2017	SH	I	Holstein	2–4	200	10	–	5%

The samples mean the total amounts of samples collected from each region

Numbers in Ne-PCR and Cq-PCR mean the positive samples identified by each method

*Ne-PCR* nested PCR, *Cq-PCR* CoCoMo-qPCR-2

### Phylogenetic analysis of the BLV-env gp51 region

**Fig. 2:**
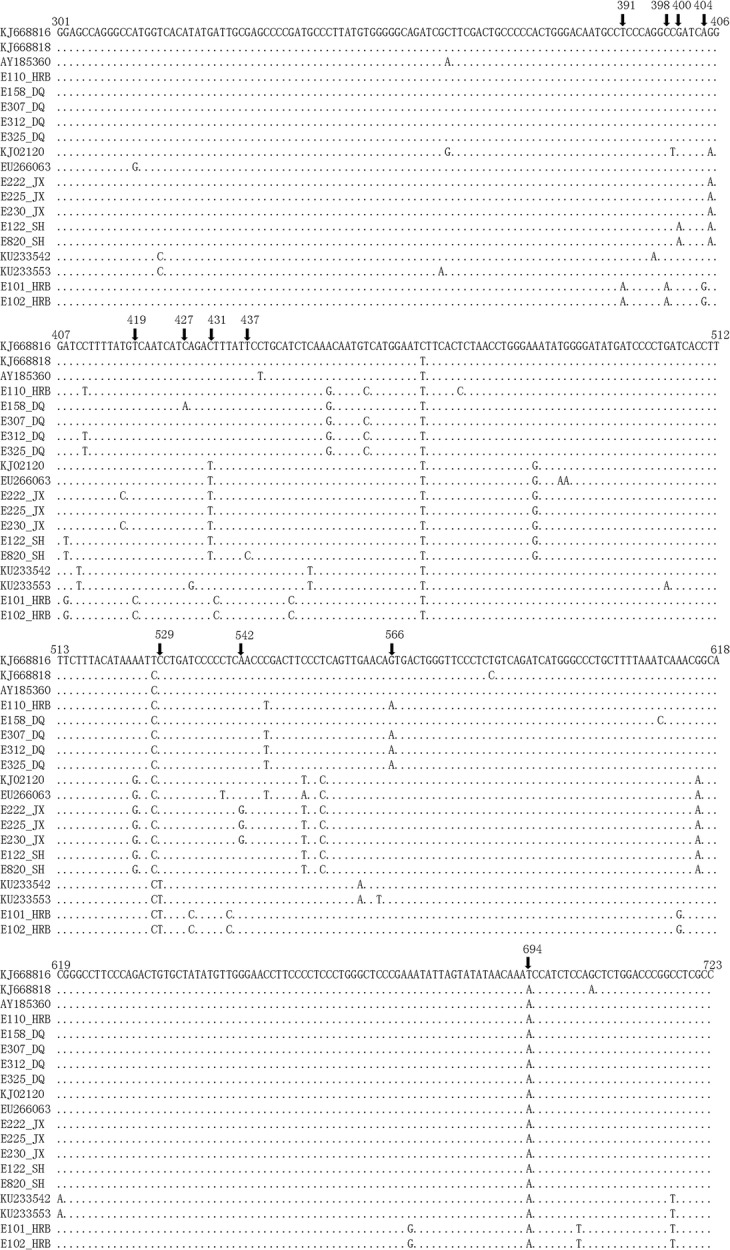
Polymorphism analysis of HLJ BLV isolates aligned with a Japanese strain, CAM69 (KJ668816.1). The sequences of our isolates were deposited in GenBank. The arrows indicate nonsynonymous nucleotide substitutions

Based on the nested PCR results, we performed a sequencing analysis. Here, we used the 423-bp gene of gp51 (derived from nested PCR-acquired 600 bp fragments) for the downstream phylogenetic analysis. To construct a phylogenetic tree, we acquired 60,423-bp gp51 sequences that were deposited in GenBank, including the currently known 10 genotypes of BLV. In Fig. 2, the alignment is shown between our isolates and G6 CAM69 (GenBank accession# KJ66816.1) [28]. In this alignment, we identified 36 substitutions in our obtained nucleotide sequences. Among these substitutions, twelve were nonsynonymous, and the others were silent. The twelve nonsynonymous substitution sites included residues 131 (nt 391), 133 (nt 398), 134 (nt 400), 135 (nt 404), 140 (nt 419), 143 (nt 427), 144 (nt 431), 146 (nt 437), 177 (nt 529), 181 (nt 542), 189 (nt 566), and 232 (nt 694).

**Fig. 3:**
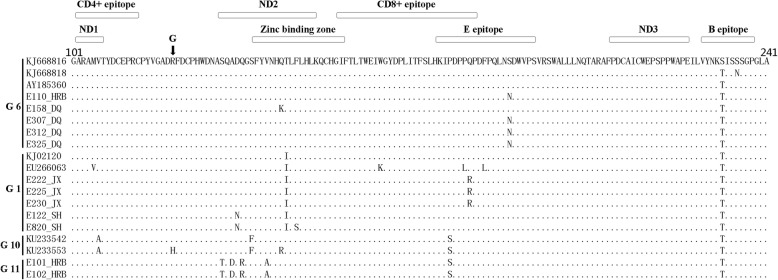
Deduced amino acid analysis of the 423-bp *env* gene of HLJ BLV isolates. The major epitope regions are shown. ND indicates the neutralizing domain. The ZB is also indicated

A further analysis of these nonsynonymous substitution sites indicated that they were distributed in several domains of BLV gp51. As shown in Fig. 3, in alignment with CAM69, we found eight substitutions in the neutralizing domain 2 (ND2) (131–149), four in the zinc-binding region (ZB) (137–155), three in the CD8+ T-cell epitope region (154–182), three in the E-epitope region (175–194), and one in the B-epitope region (228–238). Substitutions in these epitope regions indicated that BLV underwent intense immune pressure in vivo. In addition to these substitution sites, conserved regions of our isolates were observed in neutralizing domain 1 (ND1), CD4+ T-cell epitope region, and neutralizing domain 3 (ND3), which could indicate their critical importance in viral fitness cost. Notably, two N-linked glycosylation sites were located in the gp51 423-bp region (N129KS and N230KS/T).

**Fig. 4:**
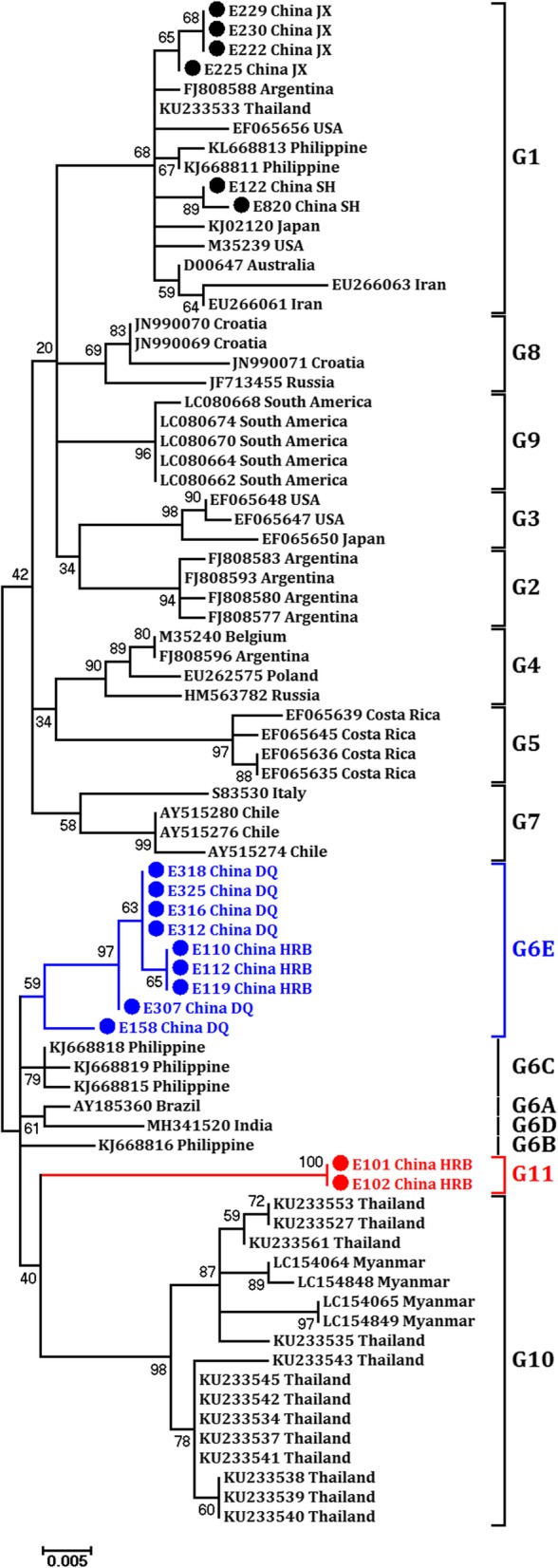
Phylogenetic analysis of BLV *env* 423-bp nucleotide sequences from China and other countries. The phylogenetic tree was made using the maximum likelihood method. The ten known major genotypes are labeled G1 to G10. The new G11 and G6E are indicated as red circles and blue circles, respectively. HLJ BLV isolates were clustered into three genotypes, G1, G6, and G11

Dependent on the phylogenetic analysis, our isolates clustered into three clades (Fig. 4). For instance, the isolates from JX and SH grouped into G1 (E122, E222 and E225, E229, E230 and E820), and the isolates from DQ (E158, E307, E312, E316, E318, E325 and E329) and HRB (E110, E112 and E119) clustered into G6. The isolates derived from DQ had slight differences from the known G6 subgenotypes A, B, C, and D. Thus, we clustered these isolates into a new subgenotype, G6E. However, two isolates (E101 and E102) from HRB were distinguished from the currently known BLV isolates and clustered into an independent branch. Here, we defined these two isolates as a new genotype, G11, adding to the members of the BLV genotype family. Thus, both G6 and G11 existed in the HRB region. In fact, we obtained isolates E101, E102, and E110 from the same herds, which indicated that multiple genotypes of BLV existed within one farm. Unlike in HRB, the isolates from JX, DQ, and SH were derived from a single “parental” strain (Fig. 4), which indicated a stable spread of BLV transmission.

### Analysis of gag alignment

Based on the nested PCR results, we further acquired the gag genes from several BLV-positive samples. As shown in Table 2, 14 substitution sites in the Gag region were found in our local strains and aligned with the G1 strain (GenBank accession# EF600696, one FLK-BLV subclone). Substitution sites were distributed in the following regions: seven in the MA region, including residues 35, 48, 63, 69, 87, 88, and 90; five in the CA region, consisting of residues 164, 171, 315, 318, and 323; and two in the NC region, including residues 329 and 365. The alignment of Gag in the listed strains showed that the MA region displayed a relatively high ratio of variation, and variations in the CA region were not prone to occur. This case should be partially attributed to the dependence on CA to maintain viral core stability. Unlike BLV gp51, no N-linked glucosylation sites were found in our acquired Gag region.

**Table 2:** Polymorphism analysis of BLV Gag region

Isolate	Viral gene	MA										CA						NC			
		2	3	4	6	6	6	8	8	9	1	1	1	2	3	3	3	3	3	3	3
		2	5	8	1	3	9	7	8	0	0	6	7	4	1	1	2	2	6	6	7
											8	4	1	9	5	8	3	9	5	9	9
EF600696-USA-G1		L	S	H	G	T	R	D	G	A	V	E	A	P	A	V	V	G	A	T	P
Gag-China-P1-G1			N	Y		I	K		E				V		V	T	I	W	T		
Gag-China-P2-G1								E		T						M	I				
Gag-China-P3-G1								E				K				M	I				
FJ914764-Argentina-G2				Y	S									S		I	I		T		L
AF033818-USA-G4		R				A	K		E		I						I		T		
LC080656-Paraguay-G6				Y		V	K		E							T	I		T		
LC080660-Bolivia-G9					S					T						I	I		T		
LC154849-Myanmar-G10				Y	S	V	K		E								I			A	

The USA strain EF600696 was used as a reference for the Gag alignment

*MA* matrix, *CA* capsid, *NC* nucleocapsid

## Discussion

In this study, we initially investigated the general status of BLV epidemics in partial regions of HLJ, China. The nested PCR was stable and reliable in our investigation (matched well with our preliminary serological test of BLV gp51). However, the CoCoMo-qPCR-2 was verified to be more sensitive in monitoring BLV-infected individuals (12.5% versus 10% in the nested PCR assay). Therefore, the CoCoMo-qPCR-2 should be considered a priority for subsequent BLV proviral DNA detection.

Our results confirmed that 68 of 730 samples were identified as BLV-positive, with an overall infection rate of 9.3%. However, it was indicated that BLV epidemics displayed regional differentiations in HLJ. The BLV infection rate in JX was the highest (31%) and that of DQ, HRB, and SH was lower, whereas QQHR and MDJ showed null BLV infection. A previous study also reported null BLV infection in QQHR [5], which was in accordance with our results. Notably, because only 100 samples in QQHR (in both Yang et al [5] and our assay) and 60 samples in MDJ were collected, monitoring more samples would further confirm these cases. If these cases are confirmed, then the low BLV infection status in both QQHR and MDJ should provide a good reference for us to control BLV transmission of other regions. In the HLJ territory, DQ, SH, and HRB were adjacent to each other and far from JX. However, the isolates of SH and JX clustered into one clade, whereas DQ and HRB isolates clustered into other clades, which could suggest a lack of correlation among the BLV genotypes with regional adjacency. Further investigation is needed to confirm whether SH retained the same viral clade as that in both DQ and HRB. Cattle in JX have been bred for several decades within farms and not often imported. Thus, the high infection rate of these cattle could be derived from the low management of breeding measures, which indicates the necessity to strengthen herd control and periodic monitoring. Currently, no effective vaccines exist to control BLV infection and transmission. Therefore, taking measures to monitor and isolate BLV-infected individuals (especially individuals with high viral loads [37]) is necessary to prevent further spread, either horizontally or vertically.

In this research, we identified a new BLV genotype, G11, by the maximum likelihood algorithm method. In fact, the neighbor-joining method was also performed to conduct the phylogenetic analysis and cluster the G11 isolates into a unique branch (data not shown). The amino acid alignment presented more information about the new G11 isolates. In the ND2/ZB, when aligned with those of G1-G10, we identified a particular motif in our two G11 isolates (Fig. 3). We observed that the clustered variation sites T-X-D-X-R-XXXX-A (T131/D133/R135/A140) together were unique in G11 and absent in the GenBank-recorded G1-G10 BLV env sequences (Additional file 1: Figure S1). The precise factors that led to this unique motif in G11 were unclear. Regardless, this motif was located in the ND2 domain and thus was possibly related to the host humoral immune responses in vivo. This motif indeed defined the genotypical features of the new G11. A recent report indicated that several residues of gp51 were under positive selection, including residues 48, 74, 82, 133, 142, and 291 [38], which could be correlated with viral immune escape in vivo. However, in our present study, only the variation site of residue 133 was monitored, which was located within the G11 T-X-D (133)-X-R-XXXX-A motif. Considering these characteristics of G11, we thus aimed to test the G11 Env infectivity. Unfortunately, we failed to acquire the G11 full-length env gene, which blocked us from further confirming its infection capacity. G11 isolates E101/E102 and G6 isolates E110/E112/E119 were derived from the same farm but diverged into two genotypes. By aligning E101 and E110 amino acid sequences, as shown in Fig. 3, we could readily identify a variation in the four residues in the G11 ND2/ZB and one residue in the G6 E-epitope region. Theoretically, a recombination potential exists between the differential G6 and G11 isolates, for both genotypical isolates coexisted on one farm. However, we still have not identified isolates that inherit the variation sites of both G6 and G11 together. Provided that recombination between G6 and G11 occurs, such an event may give rise to new BLV genotypes or subgenotypes, which is an interesting issue for further study.

BLV genotypes have been reported in countries near HLJ, China. For instance, G1, G5, and G7 were prevalent in Mongolia [30]; G1 and G3 were found in Korea [29]; and G1, G3, and G5 were circulating in Japan [20, 24]. Our results showed that BLV was clustered into three clades in HLJ, including G1, G6, and G11. G1 was prevalent in JX and SH, and G6 existed in DQ, whereas G6 and G11 were circulating in HRB. Notably, G6 and G10 were reported to be prevalent in the yaks of southwestern China [36]. Therefore, to date, G1 is currently the dominant genotype in HLJ, China, and surrounding countries, and G6 appeares in both southwestern and northeastern China. Determining whether G6 is prevalent all over China also deserves further exploration.

Envelope glycosylation is believed to be critical in viral immune evasion [39, 40]. Recently, de Brogniez Alix et al. confirmed that N-linked glycosylation of the BLV SU protein could affect its pathogenicity and found that N129 was a probable glycosylation site [41], which was beneficial for viral escape from neutralization [42]. In the variation analysis of our isolates, the N129 glycosylation site in the ND2 region was not affected by the NAS to NAT substitution. In addition, a nonsynonymous substitution of the N-linked glycosylation site (NKT to NKS) has also been detected in the B-epitope region (N230, a critical glycosylation site indicated by de Brogniez Alix et al [41]). Overall, these conserved N-linked glycosylation sites were unaffected, probably due to their importance in biological functions in vivo. In the ZB, the cysteine (residue 152) in the motif FLHLKQCHGI was critical for zinc-ion binding [43]. We also observed this conserved site in our isolates, although other variation sites also existed within the ZB. In addition to the ND2/ZB, we also detected variations in the CD8^+^ T-cell epitope/E-epitope region and the B-epitope region, which were similar to previous observations of substitutions in these epitope regions [27, 38, 44]. Unlike the gp51 region, the BLV p24 region showed a relatively conserved feature, which could facilitate the development of BLV assay kits and vaccine candidates.

## Conclusions

BLV infection of Dairy cattle was common in major regions of HLJ, and the infection rate was different among these regions. Three genotypes, G1, G6, and the newly identified G11, are currently circulating in HLJ, northeastern China. Such information provides a good reference for later investigations of BLV infection in dairy cattle of HLJ and China.

## Materials and methods

### Sample collection and DNA extraction

Blood samples were taken from cattle at nine different farms distributed in six major regions of HLJ, including Qiqihar (QQHR), Daqing (DQ), Suihua (SH), Harbin (HRB), Mudanjiang (MDJ), and Jixi (JX) (Fig. 1b). As listed in Table 1, a total of 730 samples were collected and examined. Each blood sample was used for subsequent DNA extraction. Genomic DNA was extracted from 200 μL aliquots using the Qiagen DNA extraction kit (QIAamp DNA Mini Kit, Hilden, Germany) following the manufacturer’s protocol and subjected to downstream nested PCR assay.

### BLV nested PCR and CoCoMo-qPCR-2

The nested PCR assay was performed to detect the BLV proviral DNA and obtain the BLV env fragments using PrimeSTAR (HS) DNA polymerase (Takara, Japan). The primers used in this study are listed in Table 3. The first-round PCR program was as follows: 98 °C for 3 min, 35 cycles of 98 °C for 15 s, 50 °C for 20 s, and 68 °C for 1 min, followed by 68 °C for 5 min. The second-round PCR conditions were the same, but internal primers were used (Table 3). The PCR products were then agarose-gel purified (gel-purification kit, Omega) and sequenced (Sangers’ analysis, Comate company, Changchun, China). The env-product size was approximately 600 bp, 423-bp fragment of which was used for phylogenetic analysis. The CoCoMo-qPCR-2 assay, as described previously [28, 45, 46], was applied to confirm its efficacy in checking the existence of BLV proviral DNA. Briefly, the primers and the probe aiming at the BLV long terminal repeat (LTR) region were synthesized (Table 3). LA-Taq polymerase (Takara, Japan) was used in this amplification. The CoCoMo-qPCR-2 program was performed as follows: 94 °C for 3 min, 35 cycles of 94 °C for 15 s, 60 °C for 30 s, followed by 60 °C for 5 min. In addition, a pair of primers was designed to obtain the BLV gag sequences (Table 3). PrimeSTAR (HS) DNA polymerase was used in the system. The PCR program for gag amplification was as follows: 98 °C for 3 min, 30 cycles of 98 °C for 15 s, 60 °C for 30 s, and 68 °C for 2 min, followed by 68 °C for 5 min. The size of the gag fragment was approximately 1.9 kb, and the fragment was subjected to a downstream sequencing analysis.

**Table 3:** Primers and probe used in this study

Gene	Round	Primer/Probe	Sequence	Ref.
Env	1st	Env-EF	5′-ATGCCYAAAGAACGACGG-3′	[23]
		Env-ER	5′-CGACGGGACTAGGTCTGACCC-3′	
	2nd	Env-IF	5′-TCTGTGCCAAGTCTCCCAGATA-3′	[20]
		Env-IR	5′-AACAACAACCTCTGGGAAGGGT-3′	
LTR	1st	CoCoMo-F	5′-AATCCMNMYCYKDAGCTGCTGAYYTCACCT-3′	[28]
		CoCoMo-R	5′-TTGCCTTACCTGMCSSCTKSCGGATAGCCGA-3′	
		LTR-probe	5′-FAM-CTCAGCTCTCGGTCC-NFQ-MGB-3′	[47]
Gag	1st	Gag-F	5′-ATGGGAAATTCCCCYTATAA-3′	This study
		Gag-R	5′-CTAGTTTAAAGGGAATTAG-3′	

Round means the round of PCR reactions

### Phylogenetic tree construction of BLV env and alignment of the Gag region

Both the BLV env and gag nucleotide sequences were edited using the Lasergene DNAStar program 6.0 (DNAStar, Madison, WI, USA). The Megalign program was used to perform pairwise and multiple alignments of the DNA sequences and deduced amino acid sequences. Approximately 70 GenBank-published sequences were used for alignment and phylogenetic tree construction. The phylogenetic analysis was conducted via MEGA6 using the maximum likelihood method and the Kimura 2-parameter model. The bootstrap value was 1000 replicates. The sequences of isolates obtained in this study were deposited in GenBank under accession numbers KU764746-KU764766, KY421037-KY421038, and MG800835-MG800837.

## Additional files

Additional file 1: **Figure S1.** Deduced amino acid alignment of G11 E101 and E102 with current known G1 to G10 isolates. E101- and E102-deduced amino acids were aligned with those of 10 known genotypes. The variation sites T (131), D (133), R (135), and A140 together (located in the ND2 domain and ZB) were highly specific in G11 E101 and E102 but not in isolates of other genotypes. (PPTX 353 kb)

## Abbreviations

BLV: Bovine leukemia virus
env: Envelope
G: Genotype
ND: Neutralizing domain
nt: Nucleotide
SU: Surface
ZB: Zinc binding
