# Supplementary figures and images for: Genotyping bovine leukemia virus in dairy cattle of Heilongjiang, northeastern China
Source: BMC Vet Res. 2019 May 29;15:179. doi: 10.1186/s12917-019-1863-3 (PMC6542110; doi:10.1186/s12917-019-1863-3)

## Slide 1
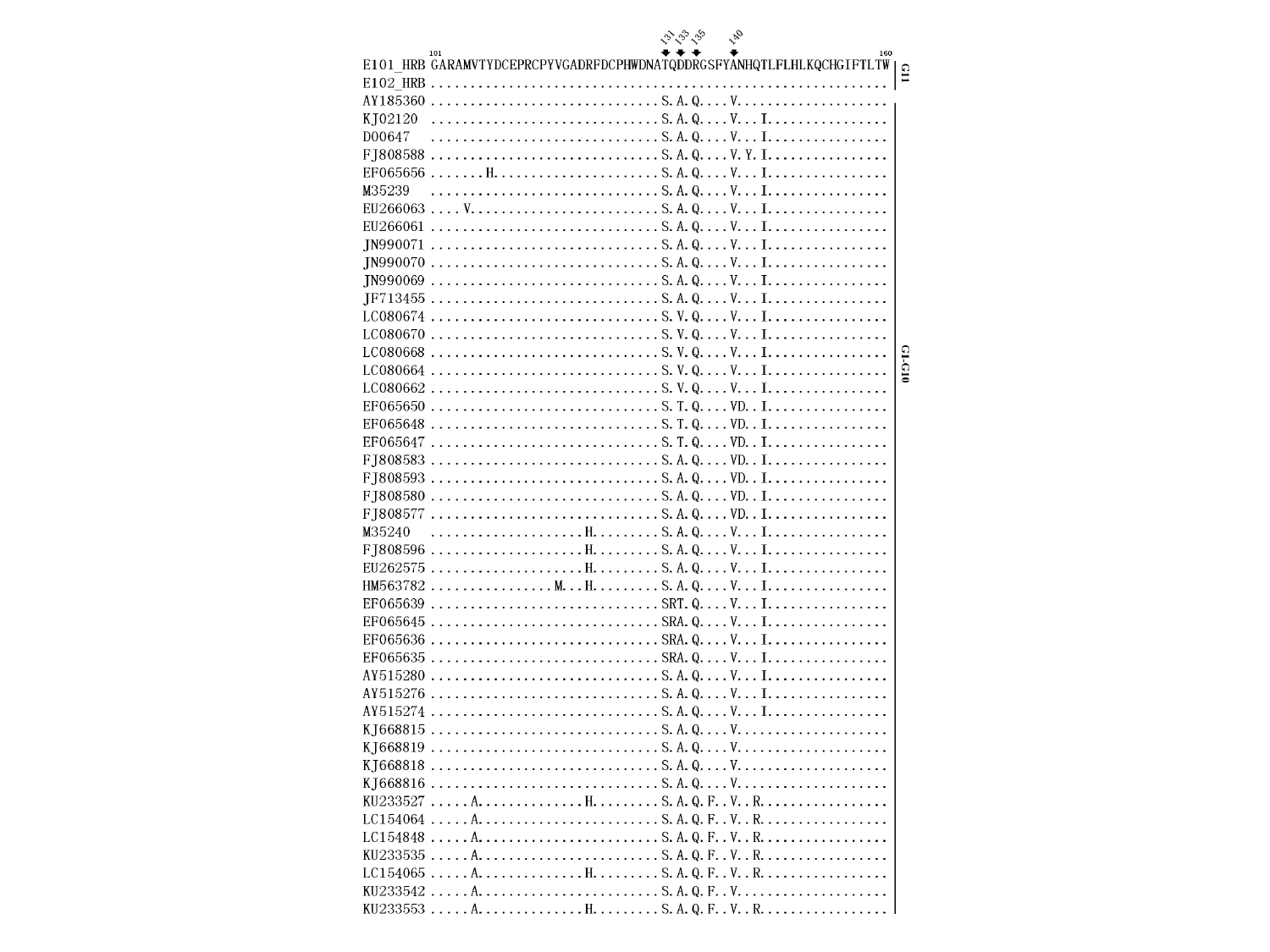

Supplement: Supplementary file 1 — Figure S1. Deduced amino acid alignment of G11 E101 and E102 with current known G1 to G10 isolates. E101- and E102-deduced amino acids were aligned with those of 10 known genotypes. The variation sites T (131), D (133), R (135), and A140 together (located in the ND2 domain and ZB) were highly specific in G11 E101 and E102 but not in isolates of other genotypes. (PPTX 353 kb) [file 12917_2019_1863_MOESM1_ESM.pptx]
